# Role of Curcuma longae Rhizoma in medical applications: research challenges and opportunities

**DOI:** 10.3389/fphar.2024.1430284

**Published:** 2024-08-06

**Authors:** Peng Zhang, Hong Liu, Yuan Yu, Shiyang Peng, Shaomi Zhu

**Affiliations:** School of Medical and Life Sciences, Chengdu University of Traditional Chinese Medicine, Chengdu, Sichuan, China

**Keywords:** Curcuma longae Rhizoma, curcumin, curcumol, β-elemene, curdione, pharmacological activities

## Abstract

Curcuma longae Rhizoma, commonly known as turmeric, is extensively utilized not only in Traditional Chinese Medicine (TCM) but also across various traditional medicine systems worldwide. It is renowned for its effectiveness in removing blood stasis, promoting blood circulation, and relieving pain. The primary bioactive metabolites of Curcuma longae Rhizoma—curcumin, β-elemene, curcumol, and curdione—have been extensively studied for their pharmacological benefits. These include anti-tumor properties, cardiovascular and cerebrovascular protection, immune regulation, liver protection, and their roles as analgesics, anti-inflammatories, antivirals, antibacterials, hypoglycemics, and antioxidants. This review critically examines the extensive body of research regarding the mechanisms of action of Curcuma longae Rhizoma, which engages multiple molecular targets and signaling pathways such as NF-κB, MAPKs, and PI3K/AKT. The core objective of this review is to assess how the main active metabolites of turmeric interact with these molecular systems to achieve therapeutic outcomes in various clinical settings. Furthermore, we discuss the challenges related to the bioavailability of these metabolites and explore potential methods to enhance their therapeutic effects. By doing so, this review aims to provide fresh insights into the optimization of Curcuma longae Rhizoma for broader clinical applications.

## 1 Introduction

Traditional Chinese Medicine (TCM) has played a pivotal role in health maintenance and disease treatment for thousands of years (Wang et al., 2021). Recognized for its holistic approach, TCM emphasizes the balance between body, mind, and environment, and utilizes a wide range of herbal formulas to treat a myriad of ailments (Matos et al., 2021). In recent years, the integration of traditional remedies with modern medical practices has gained considerable attention globally. This resurgence is driven by a growing body of scientific research that validates the efficacy of many traditional botanical drugs through modern pharmacological insights (Xu et al., 2021). As the world leans towards more natural and preventive health measures, the medicinal potential of TCM botanical drugs presents promising prospects for developing innovative therapeutic solutions that address complex health issues (Zhang X. et al., 2021).

Curcuma longae Rhizoma, also referred to as “Ezhu” in China and commonly known as “turmeric”in English, is accurately identified as Curcuma longa L. [Zingiberaceae; Curcumae longae rhizoma] in pharmacopeia (Zhang et al., 2017). While it is widely used in traditional Chinese medicine, its medicinal uses extend across various traditional medicine systems in Asia, such asc and it is now utilized globally (Ayati et al., 2019). The botanical drug primarily grows in tropical and subtropical regions, including Thailand, Indonesia, and Malaysia. In China, approximately 20 varieties of Curcuma Rhizoma are cultivated, mainly in provinces such as Sichuan, Fujian, Zhejiang, and Guangxi, making the raw materials readily available (Liu et al., 2014).

**FIGURE 1:**
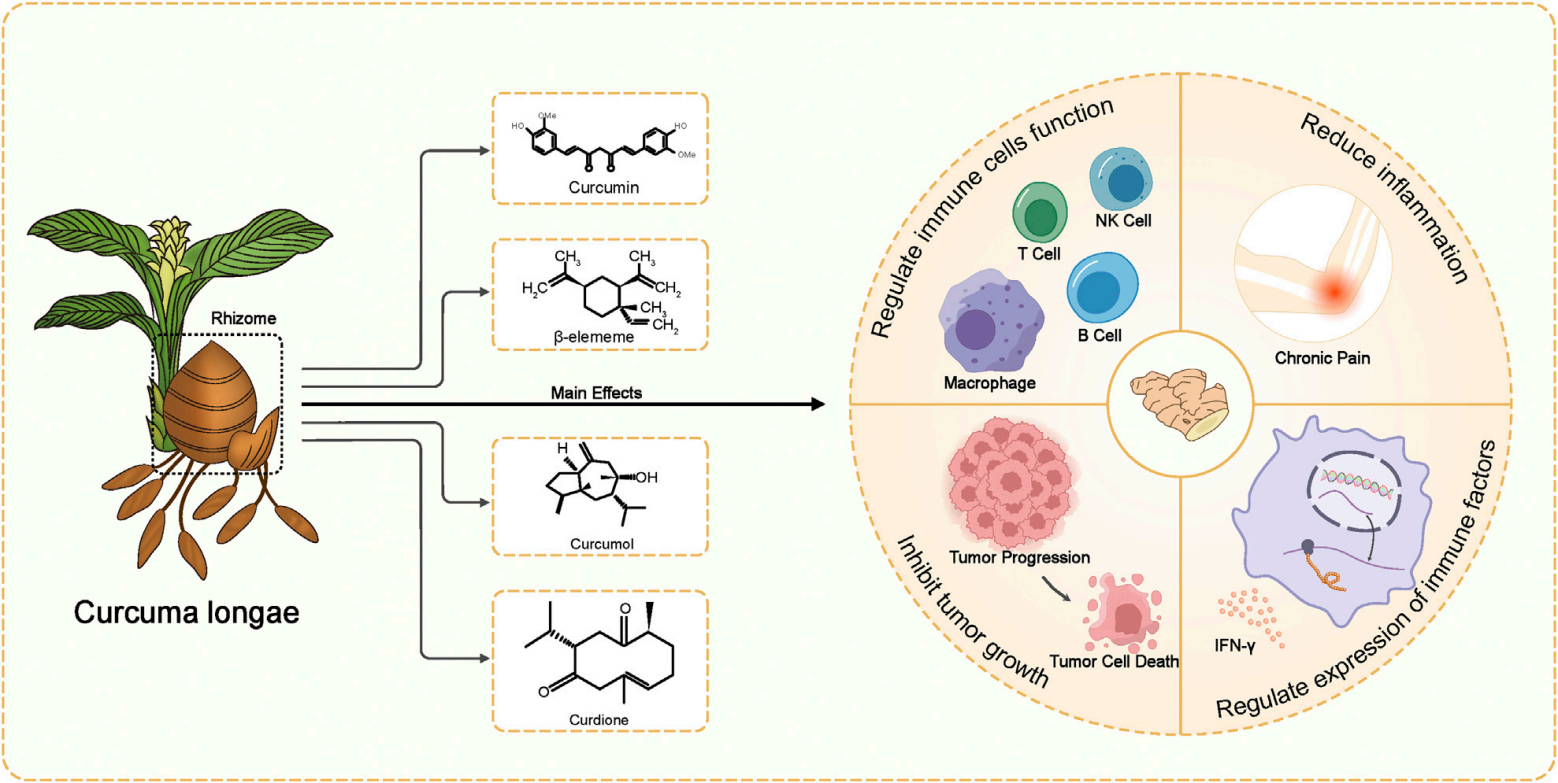
Main ingredients of Curcuma longae Rhizoma and their pharmacological effects.

From the perspective of traditional Chinese medicine, Curcuma longae Rhizoma is valued for its ability to activate blood, reduce accumulation, promote qi circulation, and relieve pain. Western medicine has recognized its broader pharmacological effects, including immune regulation, anti-thrombotic, anti-inflammatory, analgesic, antiviral, anticancer, neuroprotective, and antioxidant properties (Lu et al., 2012; Qin et al., 2017; Rahaman et al., 2020; Zhang et al., 2023; Zhao et al., 2023) (Figure 1). Due to these benefits, it is frequently used to treat conditions like tumors, diabetes, and neurological and mental diseases (Sharifi-Rad et al., 2017). Various pharmaceutical formulations such as Curcuma injections, eye drops, creams, microspheres, and suppositories have been developed and are currently used in clinical practice.

**FIGURE 2:**
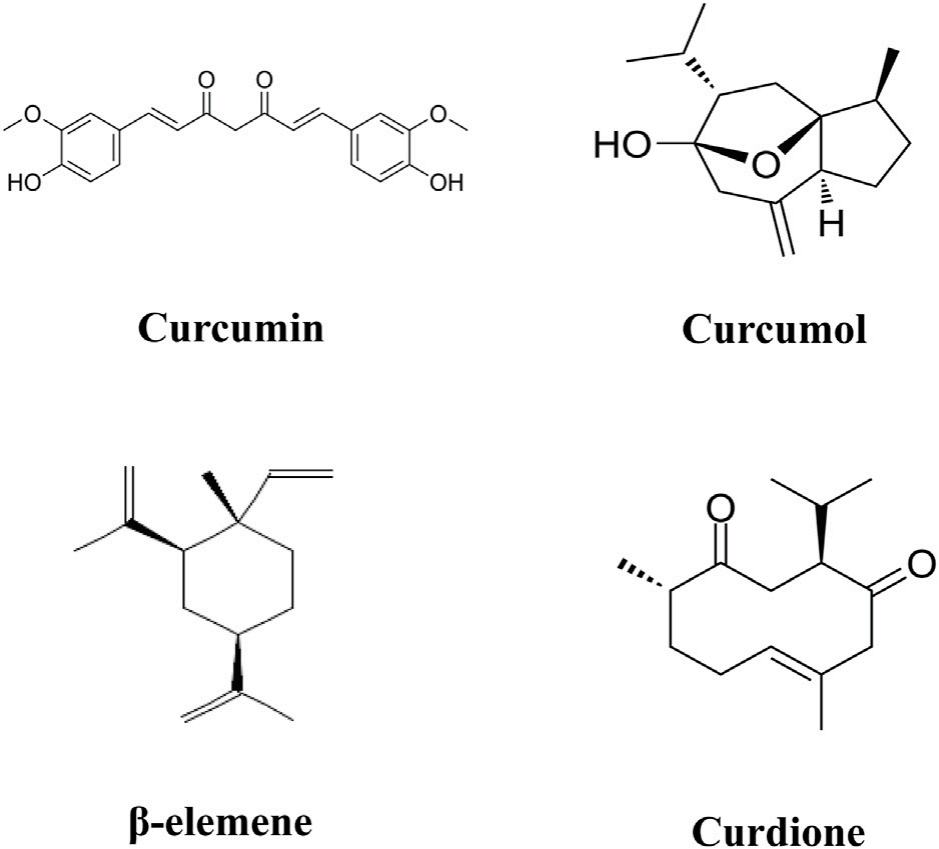
Chemical structures for curcumin, curcumol, β-elemene, and curdione.

The advancement of modern pharmacological analysis technologies has significantly propelled research into the therapeutic mechanisms of Curcuma longae Rhizoma. Although various studies have optimized the extraction of multiple bioactive metabolites using techniques like ultrasonic-assisted extraction and dual-wavelength gradient elution high-performance liquid chromatography (Shirsath et al., 2017; Wang et al., 2017), comprehensive data analysis on these metabolites remains scarce. Known bioactive metabolites such as curcumin, β-elemene, curcumol, and curdione (Figure 2) function through diverse targets and mechanisms. To foster the development and ensure the safe clinical use of Curcuma longae Rhizoma, this review compiles recent advancements in the primary clinical therapeutic effects of these active metabolites, their action mechanisms, and strategies to enhance bioavailability. Our study is innovative in its in-depth exploration of how the main active metabolites of turmeric interact with molecular targets and signaling pathways like NF-κB, MAPKs, and PI3K/AKT. We also address the challenges associated with the bioavailability of these metabolites and propose methods for their enhancement. Furthermore, this review dissects the multiple mechanisms by which Curcuma longae Rhizoma achieves therapeutic outcomes in various clinical settings, aiming to broaden its clinical application strategies. This contrasts with existing literature that often focuses on single medicinal botanical drugs or combinations, emphasizing morphological, compositional, and pharmacological activities (Wu and Tong, 2022; Zhu et al., 2023; Lin et al., 2024). In comparison, our comprehensive evaluation provides insights into synergistic effects among active metabolites, offering innovative strategies for optimizing clinical applications of Curcuma longae Rhizoma.

## 2 Pharmacological effects of Curcuma longae Rhizoma

### 2.1 Anti-inflammatory effect

The extensive interaction of Curcuma longae Rhizoma with multiple inflammatory pathways not only highlights its efficacy as an anti-inflammatory agent but also its potential in the treatment of various inflammatory-related conditions. This includes chronic inflammatory diseases, autoimmune disorders, and conditions associated with metabolic syndrome where inflammation plays a critical role.

The anti-inflammatory properties of Curcuma longae Rhizoma are largely attributed to its impact on crucial biochemical pathways that regulate inflammation in the human body (Rahaman et al., 2020). The primary mechanism involves the inhibition of the Nuclear Factor kappa-light-chain-enhancer of activated B cells (NF-κB) pathway. Normally inactive in the cytoplasm and bound to the inhibitory protein IκB, NF-κB becomes active when IκB degrades, facilitating its translocation to the nucleus where it promotes the expression of pro-inflammatory genes (Buhrmann et al., 2021). Curcumin, a major active metabolite in Curcuma longae Rhizoma, effectively blocks this process by preventing IκB degradation, thereby reducing the expression of pro-inflammatory cytokines such as TNF-α, IL-1, and IL-6 (Edwards et al., 2020).

Additionally, Curcuma longae Rhizoma modulates other significant inflammatory pathways including the Cyclooxygenase-2 (COX-2) and Lipoxygenase (LOX) enzymes that catalyze the production of major inflammatory mediators like prostaglandins and leukotrienes (Cho et al., 2009). By inhibiting these enzymes, the production of these mediators is decreased, contributing to reduced inflammation. Curcuma longae Rhizoma also influences the Mitogen-Activated Protein Kinases (MAPKs) and Phosphoinositide 3-kinases (PI3K)/Akt pathways, both known for their roles in cellular responses to inflammation (Guo et al., 2023). The botanical drug’s active metabolites dampen the activation of these pathways, further alleviating inflammatory responses at the cellular level.

### 2.2 Anti-oxidant effect

Curcuma longae Rhizoma is highly regarded for its robust antioxidant properties, largely attributed to its active metabolite, curcumin or β-elemene, and other phytochemicals present in the rhizome (Burapan et al., 2020). These metabolites are effective in neutralizing free radicals—unstable molecules that cause oxidative stress and cellular damage, contributing to aging and various chronic diseases, including cardiovascular disorders, diabetes, and neurodegenerative diseases (Herisman et al., 2022).

The antioxidant mechanisms of Curcuma longae Rhizoma are multifaceted. Primarily, curcumin directly scavenges different forms of free radicals such as reactive oxygen and nitrogen species (Maithilikarpagaselvi et al., 2020). Beyond direct scavenging, Curcuma longae Rhizoma significantly enhances the body’s antioxidant defenses. It upregulates the expression of key endogenous antioxidant enzymes, including glutathione peroxidase, superoxide dismutase (SOD), and catalase. These enzymes are crucial for the detoxification of harmful oxidants within the body (Lukitaningsih et al., 2019).

Additionally, Curcuma longae Rhizoma modulates several critical signaling pathways associated with oxidative stress and inflammation. One pivotal pathway is the Nuclear Factor Erythroid 2–Related Factor 2 (Nrf2) pathway, which regulates the expression of detoxifying enzymes and antioxidant proteins (Serafini et al., 2019). Curcumin promotes the dissociation of Nrf2 from its inhibitor, Keap1, allowing Nrf2 to translocate to the nucleus where it enhances the transcription of genes encoding antioxidant proteins and phase II detoxification enzymes (Khin Aung et al., 2022). This not only boosts the cell’s innate resilience against oxidative damage but also enhances its capability to respond to oxidative stressors.

### 2.3 Neuroprotective effect

Curcuma longae Rhizoma is widely recognized for its neuroprotective effects, which are pivotal in combating neurodegenerative diseases. Many active metabolites of Curcuma longae Rhizoma, such as curcumin, modulate the NF-κB pathway, significantly reducing neuroinflammation, which directly impacts the progression of neurodegenerative diseases such as Alzheimer’s and Parkinson’s (Jyotirmayee and Mahalik, 2022). By suppressing the production of inflammatory cytokines in the brain, Curcuma longae Rhizoma slows disease progression, helping manage symptoms and potentially altering disease trajectories (Lee et al., 2023). Furthermore, its potent antioxidant properties combat oxidative stress, a primary contributor to neuronal damage. The botanical drugs enhances the activity of endogenous antioxidants, shielding neural cells from oxidative damage and preventing cell death (Razavi and Hosseinzadeh, 2020).

In addition to its anti-inflammatory and antioxidant actions, Curcuma longae Rhizoma also plays a crucial role in the regulation of neurotransmitter functions. It influences key neurotransmitter systems, including dopamine and serotonin, which are essential for maintaining cognitive function and emotional balance (Abbas et al., 2020). The modulation of these neurotransmitters provides significant benefits in managing symptoms associated with neurological disorders such as depression and Parkinson’s disease (Lian et al., 2020). By influencing these neurotransmitter pathways, Curcuma longae Rhizoma contributes to better mental health and improved quality of life for individuals suffering from these conditions.

### 2.4 Anti-cancer activity

Curcuma longae Rhizoma, particularly noted for its high content of bioactive metabolites like curcumin, demonstrates significant anticancer properties (Sultana et al., 2021). These metabolites are effective in inhibiting cancer progression through multiple mechanisms: inducing cell cycle arrest at various checkpoints and triggering apoptosis in cancer cells (Li et al., 2022). This is achieved by activating caspases and downregulating anti-apoptotic proteins, thereby promoting programmed cell death. Furthermore, Curcuma longae Rhizoma metabolites inhibit angiogenesis, a critical process for tumor growth and metastasis, by interfering with angiogenic factors such as VEGF and bFGF. This limits the nutrient and oxygen supply to tumors, hampering their ability to grow and spread (Zhang Y. et al., 2021).

In addition to these direct actions against cancer cells, Curcuma longae Rhizoma’s anti-inflammatory effects play a crucial role in its anticancer activity. By modulating inflammatory pathways, such as NF-κB and COX-2, it reduces the inflammatory environment that can foster cancer progression (Xu et al., 2020). Moreover, it influences epigenetic changes, potentially reversing aberrant methylation patterns in cancer cells to restore normal cell functions (Fabianowska-Majewska et al., 2021). Given these promising mechanisms, ongoing clinical trials are essential to determine the optimal dosages and formulations for integrating Curcuma longae Rhizoma into conventional cancer treatments (Karaboga Arslan et al., 2022). The promising results from preclinical studies suggest that Curcuma longae Rhizoma could be a potent, non-toxic adjunct to existing cancer therapies, deserving further exploration and clinical testing to harness its full potential (Sultana et al., 2021).

### 2.5 Anti-microbial activity

Curcuma longae Rhizoma exhibits substantial antimicrobial properties, making it an effective natural agent against a variety of pathogens including bacteria, viruses, fungi, and parasites (Jena et al., 2020). The primary bioactive metabolite, curcumin, along with other metabolites in the rhizome, disrupts the integrity of microbial cell membranes, leading to cell content leakage and death. This mechanism is particularly effective against bacteria and fungi, compromising their essential structures and functions (Tran-Trung et al., 2023). Additionally, Curcuma longae Rhizoma interferes with viral replication processes and inhibits biofilm formation, a defense mechanism that makes pathogens resistant to conventional treatments (Shenge et al., 2021). These actions, combined with its ability to modulate the host’s immune response, enhance its efficacy in combating infections and supporting the body’s natural defenses.

## 3 Research on the regulation of various diseases by Curcuma longae Rhizoma

The extensive research on Curcuma longae Rhizoma has documented numerous bioactive metabolites, highlighting their significant regulatory potential in treating a variety of diseases. These metabolites have shown promising anti-inflammatory, antioxidant, and anti-cancer effects. However, significant limitations persist, including low bioavailability, particularly of curcumin, which complicates achieving effective therapeutic concentrations. Moreover, while mechanisms such as anti-inflammatory and antioxidant actions are known, the specific cellular interactions remain unclear. Additionally, the diversity in disease models and health conditions studied often lacks standardized research designs and uniform outcome measures, adding variability that hinders clear interpretations and comparisons. To advance the therapeutic potential of Curcuma longae Rhizoma, research must focus on enhancing bioavailability, employing rigorous standardized clinical trials, and clarifying cellular mechanisms to reliably integrate these bioactive metabolites into clinical practice.

### 3.1 Therapeutic effects and acting mechanisms of curcumin

**FIGURE 3:**
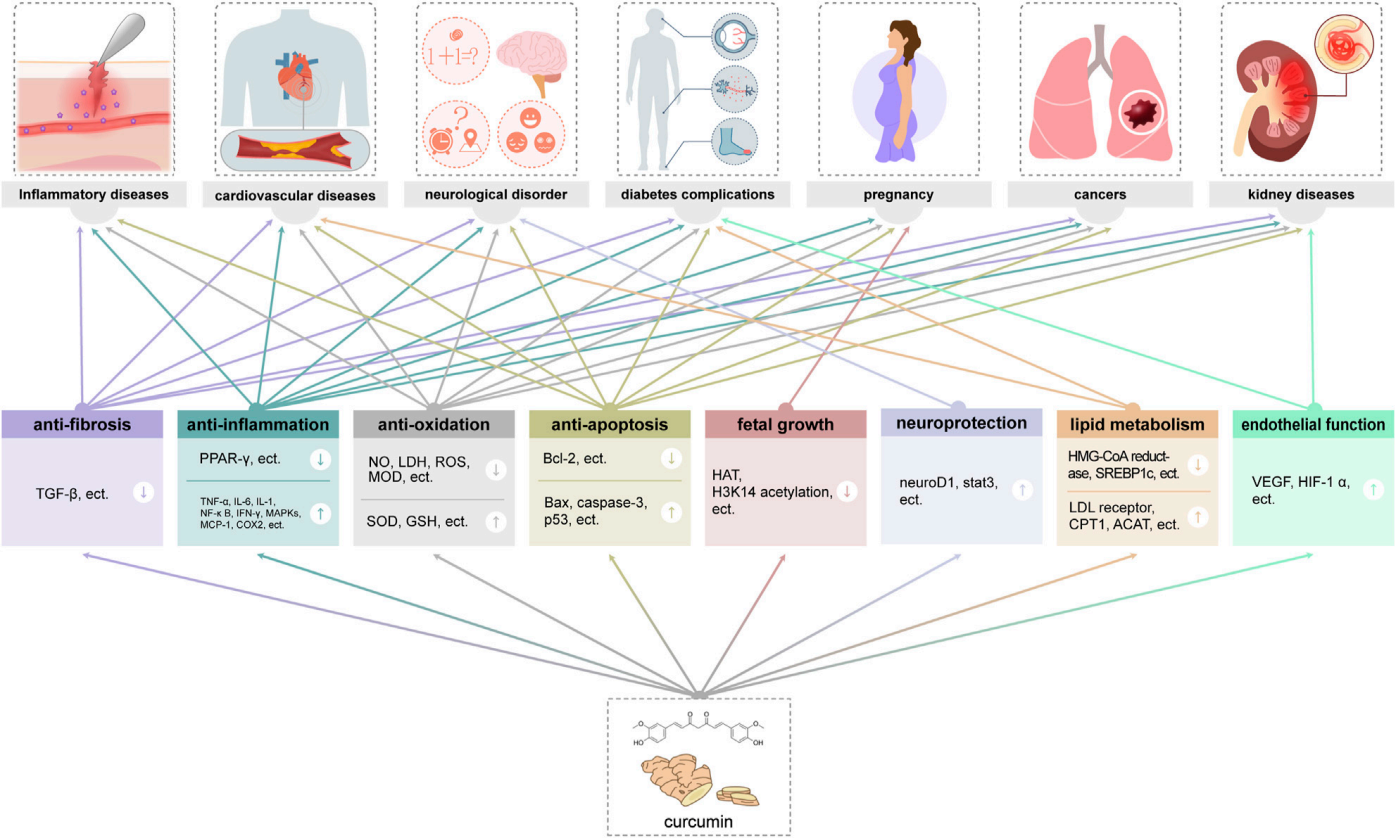
Main pharmacological and molecular targets of curcumin.

Among the active metabolites of Curcuma longae Rhizoma, curcumin was founded to obtain the most extensive coverage in clinical practice (Fuloria et al., 2022). Studies have shown that curcumin as a natural metabolite has a variety of immunoregulatory effects (Yuandani et al., 2021), and it is believed that there are various cellular pathways involved in the whole process of curcumin in neurodegenerative diseases, cardiovascular diseases, lung diseases, metabolic disorders, autoimmune diseases, malignant tumours, diabetes, and Alzheimer’s disease, etc. The antioxidant properties, affecting multiple molecular targets and signaling pathways to exert anti-tumor and immunomodulatory effects (Kahkhaie et al., 2019), and the anti-inflammatory effects of curcumin are also critical (Aggarwal and Harikumar, 2009). Curcumin can exert antioxidant effects through multiple mechanisms, including reducing free radical production, increasing antioxidant enzyme activity, and enhancing mitochondrial function. In addition, curcumin also has anti-inflammatory effects, which can play a role by inhibiting the activation of inflammatory cells and regulating inflammatory signaling pathways (Menon and Sudheer, 2007) (Figure 3).

#### 3.1.1 Anti-viral effect

Curcumin, known to activate peroxisome proliferator-activated receptor γ (PPAR-γ), plays a crucial role in modulating the cytokine storm associated with COVID-19, suggesting its potential as a therapeutic agent (Ciavarella et al., 2020). PPAR-γ, a nuclear receptor, is instrumental in regulating inflammatory and immune responses. The engagement of PPAR-γ by curcumin may represent a strategic approach to mitigate the severe inflammatory reactions observed in COVID-19 patients. Research has demonstrated curcumin’s efficacy in activating PPAR-γ, leading to reduced inflammation across various pathological conditions (de Oliveira et al., 2022). Additionally, Bormann et al. (2021) highlighted curcumin’s potential against SARS-CoV-2, where an aqueous extract from Curcuma longa L. was shown to completely neutralize the virus *in vitro* at a dilution of 1:32, without causing cytotoxic effects in Calu-3 and Vero E6 cells. Moreover, at a concentration of 14 μg/mL, curcumin significantly reduced SARS-CoV-2 RNA levels in the supernatant of these cells. These studies suggest that curcumin and the extracts from C. longa L. might interfere with the interaction between the SARS-CoV-2 spike protein and its ACE2 receptor on host cells.

Furthermore, curcumin’s therapeutic potential extends to influenza A virus (IAV). In a dose-dependent study by Dai et al. (2018), *in vitro* treatment with 150 and 50 mg/kg of curcumin resulted in decreased lung injury and reduced IAV replication. This finding was corroborated by Han et al. (2018), who administered curcumin to mice at doses of 30 or 100 mg/kg post-IAV infection, observing enhanced survival rates and diminished viral load in lung tissues. The models used in these studies provide a basis for understanding curcumin’s pharmacological effects, yet a critical analysis of their data and methodology, including control groups, duration of treatment, and extract specifics, is crucial for validating these results. Further investigations are needed to establish the full spectrum of curcumin’s safety and efficacy in humans and to delineate its potential applications in clinical settings.

In light of this evidence, while curcumin shows promise, the scientific community must engage in rigorous and critical examination of its pharmacological properties and clinical viability, considering the variability of experimental setups and the natural complexity of bioactive metabolites.

#### 3.1.2 Immune regulation in allergic disease

The immunomodulatory properties of curcumin in combating chronic allergic diseases target several molecular mechanisms. Curcumin affects transcription factors like NF-κB, MAPKs, PI3K/AKT, Wnt/β-catenin, and Nrf2, which play pivotal roles in controlling inflammatory responses (Kunnumakkara et al., 2017). It inhibits macrophage degranulation, reduces IgE-mediated allergic reactions, diminishes eosinophil infiltration, and modulates cytokine production and immune cell operations (Haftcheshmeh et al., 2022). A key clinical trial by Wu and Xiao (2016) demonstrated that a daily administration of 500 mg of curcumin over 2 months elevated IL-10 levels while reducing TNF-α, IL-8, and IL-4 production (Wu and Xiao, 2016). Similarly, Abidi et al. reported improvements in airway obstruction in asthma patients treated with the same daily dose of curcumin for a month, showcasing its potential in enhancing lung function and reducing allergic symptoms (Abidi et al., 2014). These studies employed randomized controlled trials, though the specifics of the curcumin extract and the completeness of trial protocols need further elucidation.

In an *in vitro* study, Zeng et al. (2023) assessed curcumin’s impact on IgE-mediated allergic responses by activating RBL-2H3 cells with IgE-antigen complexes. This activation induced cellular swelling, which was significantly ameliorated by curcumin at 10 μM, resulting in reduced release of β-hexosaminidase, IL-4, and TNF-α in a dose-dependent manner (Zeng et al., 2023). This confirms curcumin’s role in suppressing IgE-mediated degranulation of RBL-2H3 cells. The study utilized specific *in vitro* models and provided clear data on dose responsiveness and the minimal active concentration, which are critical for understanding curcumin’s pharmacodynamics.

While these findings underscore curcumin’s therapeutic promise, the data predominantly derive from controlled environments, with *in vivo* human studies being less prevalent. More extensive clinical trials are required to validate these effects in diverse human populations and under varying clinical conditions. Additionally, the variability in curcumin formulations used across studies raises questions about the comparability and consistency of results, suggesting a need for standardized extracts in future research.

#### 3.1.3 Effects on rheumatoid arthritis

Curcumin has shown potential therapeutic effects in rheumatoid arthritis (RA) through mechanisms that involve the modulation of inflammation and immune function. Specifically, curcumin may reduce the production of pro-inflammatory cytokines, regulate immune cell differentiation and function, and influence signaling pathways central to inflammation and autoimmunity (Mohammadian Haftcheshmeh et al., 2021). Studies have demonstrated that administration of curcumin at 80 mg/kg for 4 months increased levels of TGF-β and FoxP3, and enhanced the population of Treg cells, while also inhibiting IL-6 cytokine levels (Wu et al., 2011; Wang et al., 2015). Curcumin has been noted for its efficacy in reducing joint swelling and pain, improving physical function, and decreasing inflammatory markers, enhancing overall quality of life.

In a targeted clinical trial, Pourhabibi-Zarandi et al. (2024) investigated the effects of curcumin supplementation on total antioxidant capacity (TAC), malondialdehyde (MDA) levels, and disease activity in 48 women with RA over an 8-week period. Participants received either a 500 mg daily dose of curcumin or a placebo. The study, which employed a double-blind, placebo-controlled design, revealed that curcumin significantly increased serum TAC levels and decreased tender and swollen joint counts, pain on a visual analog scale (VAS), and Disease Activity Score-28 (DAS-28) (all *p* < 0.001), alongside a notable reduction in MDA levels (Pourhabibi-Zarandi et al., 2024).

While these findings are promising, they are primarily derived from controlled environments with specified patient groups, which may limit their generalizability. The studies predominantly focus on short-term outcomes, leaving the long-term safety and efficacy of curcumin in RA somewhat unclear. Further research is needed to confirm these results across diverse populations and to explore the pharmacokinetics of curcumin, including its bioavailability and optimal dosing strategies.

#### 3.1.4 Effects on cardiovascular disease

Curcumin is recognized for its potential therapeutic effects on cardiovascular disease, attributed to its anti-inflammatory, antioxidant, and anti-thrombotic properties. This metabolite can alleviate inflammation, oxidative stress, endothelial dysfunction, and platelet aggregation, likely by targeting molecular pathways such as NF-κB, Nrf2, AMPK, and PPAR-γ (Keihanian et al., 2018; Pourbagher-Shahri et al., 2021). Historically, curcumin has been utilized to treat various cardiovascular conditions (Li et al., 2020), and recent studies suggest it may be particularly effective in managing dyslipidemia due to its ability to modulate lipid metabolism (Peng et al., 2021).

Evidence from several randomized controlled trials indicates that curcumin significantly reduces blood lipid levels, including total cholesterol, LDL cholesterol, and triglycerides in patients at risk for cardiovascular disease (Saeedi et al., 2022). The mechanism behind these effects involves upregulating LDL receptor expression and inhibiting the activity of 3-hydroxy-3-methylglutaryl coenzyme A reductase (HMG-CoA reductase), critical for cholesterol synthesis (Qin et al., 2017). These actions are mediated through a network of molecular pathways, including the NF-κB, Nrf2, AMPK, and PPAR-γ pathways, which are essential in regulating not only lipid metabolism but also inflammation, oxidative stress, glucose metabolism, and vascular function (Zhang and Kitts, 2021).

Further supporting curcumin’s cardiovascular benefits, a randomized, double-blind, placebo-controlled trial by Dastani et al. (2023) assessed the effects of nanocurcumin (80 mg/day) in conjunction with standard pharmacotherapy on patients with mild to moderate coronary artery disease (CAD) with angiographic stenosis less than 70%. Over 90 days, treatment with nanocurcumin resulted in significant reductions in high-sensitivity C-reactive protein (hs-CRP) and lipoprotein A [Lipo(a)], indicating a decrease in inflammatory and atherogenic markers (Dastani et al., 2023). This suggests that nanocurcumin, at a daily dose of 80 mg, may help prevent the progression of atherosclerosis and potentially reduce cardiovascular events in patients with diabetic heart disease by attenuating inflammatory indices.

#### 3.1.5 Effects on diabetes

Curcumin’s potential as an anti-diabetic agent is supported by its multifaceted mechanisms, which include improving insulin sensitivity, enhancing glucose uptake and utilization, regulating lipid metabolism, and mitigating inflammation and oxidative stress. Zhao et al. (2021) noted that curcumin reduces the activity of genes involved in liver fat production, such as those coding for sterol regulatory element-binding protein 1c (SREBP1c) and carbohydrate response element-binding protein (ChREBP), which are key in cholesterol synthesis. Furthermore, curcumin boosts the activity of enzymes like carnitine palmitoyltransferase 1 (CPT1) and acyl-CoA cholesterol acyltransferase (ACAT) to enhance lipid mobilization (Zhao et al., 2021).

Curcumin also modulates lipid accumulation in the liver by activating PPAR-γ through the AMPK pathway, showcasing its antioxidant capabilities. In terms of anti-inflammatory actions, it reduces the expression of pro-inflammatory mediators such as monocyte chemotactic protein-1 (MCP-1), interleukin-1β (IL-1β), tumor necrosis factor-alpha (TNF-α), interleukin-6 (IL-6), and cyclooxygenase-2 (COX2), which are significantly elevated in type 2 diabetes (Pivari et al., 2019). Clinical studies by Panahi et al. (2018) further underscore curcumin’s therapeutic potential. In their 2018 study, a combination of curcumin (500 mg/day) and piperine significantly reduced C-peptide, HbA1c, and glucose levels, while their 2017 study indicated that higher doses of curcumin (1,000 mg) with piperine increased adiponectin levels, reduced leptin levels, and improved the leptin/adiponectin ratio (Panahi et al., 2017). These results illustrate not only the beneficial impact of curcumin on blood glucose and inflammation but also its potential to ameliorate liver markers when combined with piperine.

Despite these promising findings, the application of curcumin in diabetes treatment necessitates further exploration, particularly concerning its long-term safety, pharmacokinetics, and optimal dosing. The study by Cao et al. (2024) in a diabetic foot ulcer (DFU) rat model adds another dimension to curcumin’s potential, showing that treatment with 30 μM curcumin downregulates miR-152-3p, which activates the FBN1/TGF-β pathway. This action helps inhibit high-glucose-induced fibroblast apoptosis, promotes fibroblast proliferation and migration, and alleviates fibroblast damage, thereby accelerating wound healing in DFU rats through enhanced angiogenesis (Cao et al., 2024).

#### 3.1.6 Effects on kidney disease

Curcumin has demonstrated significant immunomodulatory roles in kidney disease across various studies, reflecting its potential in both acute and chronic renal disorders. In acute kidney injury (AKI) models, Kaur et al. (2016) observed that treatment with curcumin notably decreased plasma Myeloperoxidase (MPO) activity, thiobarbituric acid reactive substances (TBARS) levels, and superoxide anion generation, while increasing glutathione levels. This suggests curcumin’s efficacy in reducing oxidative stress markers significantly in rat models (Kaur et al., 2016). Additionally, Topcu-Tarladacalisir et al. (2016) reported that oral administration of curcumin at a dose of 200 mg/kg in Wistar albino male rats decreased levels of caspase-3, malondialdehyde (MDA), MPO, TNF-α, and various interleukins, showing protection against cisplatin-induced renal dysfunction (Topcu-Tarladacalisir et al., 2016).

In chronic kidney disease (CKD), Ali et al. (2018) demonstrated that curcumin improved renal function by reducing plasma levels of cystatin C, sclerostin, and adiponectin, while enhancing renal antioxidant enzymes such as glutathione (GSH), superoxide dismutase (SOD), Nuclear factor erythroid 2-related factor 2 (Nrf2), and catalase (CAT) (Ali et al., 2018). Hu et al. (2016) found that curcumin mitigated cyclosporin A-induced kidney disease in mice by decreasing CpG methylation in the klotho promoter, thus increasing klotho expression and inhibiting Transforming growth factor-beta (TGF-β) signaling (Hu et al., 2016).

Noonin and Thongboonkerd (2024) extended these findings by treating HK-2 cells with 625 ng/mL curcumin, which alleviated high-glucose-induced stimulation of renal fibroblast activation through the reduction of intracellular reactive oxygen species (ROS) and TGF-β secretion (Noonin and Thongboonkerd, 2024). Stankovic et al. (2020) further supported these results by demonstrating that curcumin reduced oxidative stress and enhanced antioxidant defenses in a rat model of nephrotoxicity induced by maleate treatment (Stankovic et al., 2020).

#### 3.1.7 Effect on chronic pain

Curcumin, known for its immunomodulatory properties, plays a critical role in chronic pain management by modulating inflammation, oxidative stress, and pain transmission pathways (Hasriadi et al., 2021). Its analgesic effects are particularly notable in conditions such as rheumatoid arthritis, osteoarthritis, neuropathic pain, and postoperative pain, where it improves physical function and reduces pain intensity (Sun et al., 2018). For instance, Koroljević et al. (2023) observed that a daily intake of 1,000–2000 mg of curcumin significantly decreased inflammatory biochemical markers and alleviated pain in osteoarthritis patients. This suggests a dose-dependent effect, highlighting the importance of establishing optimal dosage parameters for maximum efficacy (Koroljević et al., 2023).

Further, in a randomized controlled trial by Sedighiyan et al. (2023), 44 episodic migraine patients were divided into a nanocurcumin group (80 mg/day) and a placebo group for a 2-month period. The study measured leptin and adiponectin mRNA expression and their serum levels in isolated PBMCs using real-time quantitative PCR and ELISA at the beginning and end of the study (Sedighiyan et al., 2023). Results demonstrated that nanocurcumin significantly upregulated adiponectin mRNA and elevated its serum levels, which correlated with a marked reduction in the frequency, severity, and duration of headaches.

These studies indicate that curcumin’s therapeutic effects in pain management are mediated through its action on specific ion channels, receptors, and signaling molecules involved in pain perception. However, while these findings are promising, they also underscore the need for further research to confirm these results in larger, more diverse populations and over longer durations. Additionally, the variability in curcumin formulations used in studies (from raw extracts to nanocurcumin) raises questions about the comparability of results and the bioavailability of different formulations. These factors are critical in translating curcumin’s laboratory efficacy into clinical practice. Overall, curcumin’s broad spectrum of action offers significant potential in the treatment of not only chronic pain but also other conditions like tumors, infections, and autoimmune diseases, due to its immunomodulatory effects.

#### 3.1.8 Effects on neuroprotection

Curcumin has demonstrated neuroprotective effects in brain injury, notably after traumatic brain injury (TBI) and in neurodegenerative conditions such as Alzheimer’s disease (AD). Studies have indicated that curcumin reduces inflammation and oxidative stress, promotes neurogenesis and axonal regeneration, and influences various signaling pathways related to neuronal survival (Khayatan et al., 2022).

In TBI models, Sun et al. (2020) administered curcumin at doses of 30 and 50 mg/kg to mice, observing a significant reduction in inflammatory cytokines including TNF-α, IL-6, IL-18, and IL-1β (Sun et al., 2020). This intervention mitigated the immune response damage to the nervous system, enhancing cognitive functions, reducing brain edema, and supporting neuronal survival and regeneration. These findings suggest a dose-dependent therapeutic effect of curcumin in reducing the adverse outcomes of TBI. Beyond TBI, curcumin’s potential to slow brain aging and prevent age-related cognitive decline has been explored. Benameur et al. (2021) highlighted its immunomodulatory role in enhancing neurogenesis, synaptic plasticity, and mitochondrial function, which are pivotal in combating neurodegenerative diseases like AD. The anti-aging effects of curcumin are primarily linked to its ability to diminish inflammation and oxidative stress (Benameur et al., 2021).

In a longitudinal study by Maruyama et al. (2024), curcumin was tested in AD mouse models to evaluate its preventive effects and impact on lifespan. The study reported a survival rate of 100% in the 0.02% curcumin group and 83% in the 0.5% curcumin group, compared to 34% in the AD control group. This suggests that long-term consumption of low concentrations of curcumin may act on tau phosphorylation, reduce brain inflammation, and delay the onset of AD, thereby extending lifespan (Maruyama et al., 2024).

#### 3.1.9 Effects on improving pregnancy outcomes

Curcumin has demonstrated potential benefits for improving pregnancy outcomes through its capacity to regulate various cellular processes critical for fetal growth and development (Masella and Cirulli, 2022). Research using both animal models and human clinical trials has shown that curcumin positively impacts pregnancy by reducing preterm birth rates, enhancing fetal growth, and preventing gestational diabetes mellitus (Tossetta et al., 2021). Specifically, in a controlled study on C57BL/6 mice, curcumin administration following alcohol exposure was found to inhibit Histone acetyltransferase (HAT) activity and reduce H3K14 acetylation. This led to decreased expression of cardiac development genes EHAND and DHAND in the fetal heart, indicating a direct molecular mechanism through which curcumin may influence embryonic development. Furthermore, the study showed that oral administration of curcumin at 100 mg/kg during pregnancy reversed the acetylation of histone H3K9 near the promoter regions of caspase-8 and caspase-3, subsequently reducing the rate of preterm births (Filardi et al., 2020).

Despite these promising results, the application of curcumin in clinical settings necessitates further exploration. The experiments highlighted above were conducted under controlled conditions, and while they provide valuable insights, the translation of these findings to broader, more diverse human populations remains a challenge. Additionally, the variability in curcumin formulations, dosages, and the bioavailability in different study settings underscores the need for standardized research methodologies to better understand its therapeutic potential and limitations. The ongoing clinical trials, as summarized in Table 1, continue to evaluate the effects of curcumin on various conditions, with a focus on optimizing dosage and understanding mechanistic pathways to better leverage its benefits.

**TABLE 1:** Pharmacological parameters and mechanism of actions of curcumin.

Therapeutic effects		Experimental type	Experimental grouping and administration method	Mechanism of action	References
Anti-viral effect	SARS-CoV-2	*In vitro*	Curcumin (The aqueous extract diluted at 1:32)	Decreased cytotoxic effect on Calu-3 and Vero E6 cells	Bormann et al. (2021)
			curcumin (14 μg/mL)	Decreased SARS-CoV-2 RNA levels	
	Influenza A virus (IAV)	*In vitro*	curcumin (150 and 50 mg/kg)	Decreased lung injury and IAV replication	Dai et al. (2018), Han et al. (2018)
		*In vivo*	Curcumin (30 or 100 mg/kg)	Increased survival and lower IAV burden	
Allergic disease	Allergic rhinitis	*In vivo*	curcumin (500 mg/day)	Increase production of IL-10 Decrease the production of TNF-α, IL-8, and IL-4 Improvement in airway obstruction	Wu and Xiao (2016)
	Asthma	*In vivo*	curcumin (500 mg/day)		Abidi et al. (2014)
Rheumatoid arthritis		*In vivo*	curcumin (80 mg/kg)	Increase in the Treg population and the expression levels of FoxP3 and TGF-β Inhibit the IL-6 cytokine level	Wu et al. (2011), Wang et al. (2015)
Anti-diabetic effect	Type2 diabetes	*In vivo*	curcumin (500 mg + piperine)	Reduced C-peptide, HbA1c and glucose levels	Panahi et al. (2018), Panahi et al. (2017)
			curcumin (1,000 mg + piperine)	Increased adiponectin Reduced leptin levels Improved the leptin/adiponectin ratio	
Kidney disease	Acute kidney injury	*In vivo*	Curcumin (200 mg/kg)	Decreased levels of caspase-3, MDA, MPO, TNF-a, and interleukins (IL-10, IL-6, IL-1b)	Topcu-Tarladacalisir et al. (2016)
Chronic pain	Osteoarthritis	*In vivo*	curcumin (1,000–2000 mg/day)	Reduce inflammatory biochemical factors and relieve pain	Koroljević et al. (2023)
Neuroprotection	Traumatic brain injury (TBI)	*In vivo*	curcumin (30 and 50 mg/kg)	Reduced TNF-α and IL-1β, IL-18, IL-6	Sun et al. (2020)
Pregnancy		*In vivo*	curcumin (100 mg/kg body weight)	Inhibited HATactivity, decreased H3K14 acetylation Reverse the acetylation of histone H3K9 near the promoter region of caspase-3 and caspase-8	Filardi et al. (2020)

#### 3.1.10 Effects on inhibition of tumor

Among the treatment effects of curcumin diseases, the antitumor effect has been widely concerned. Curcumin displayed inhibitory effects on several types of cancer, such as colon cancer, liver cancer, lung cancer and breast cancer. Molecular targets of curcumin include regulation of transcription factors, enzyme activities, and signaling pathways (Shehzad et al., 2012).

**FIGURE 4:**
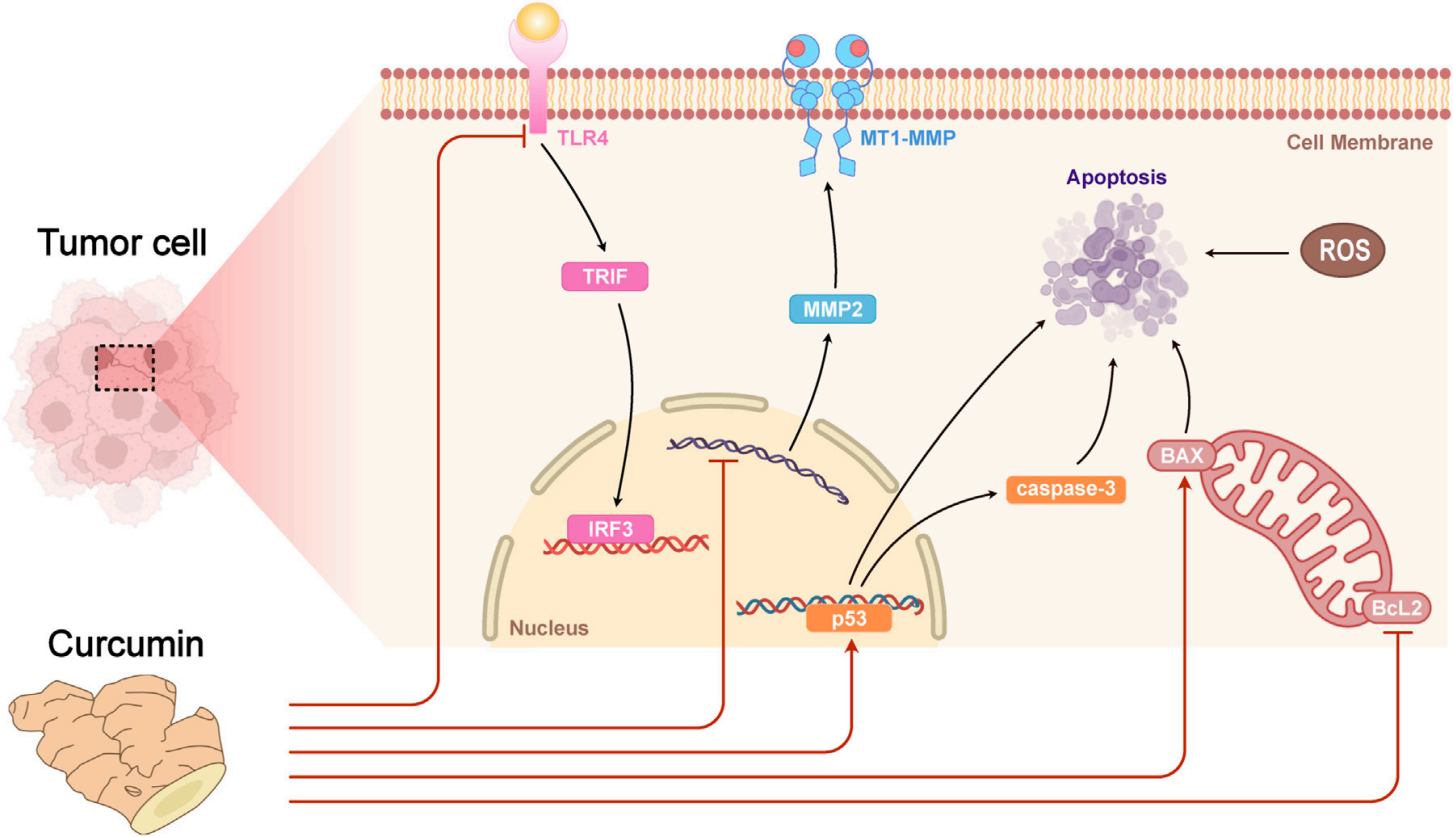
Molecule mechanism of antitumor actions of curcumin.

Curcumin can decrease the growth and metastasis of tumor cells by regulating the expression of immune-related factors such as IFN-γ, IL-2, etc (Jagetia and Aggarwal, 2007). In mouse models, curcumin can enhance the phagocytose ability of macrophages to phagocytose and the killing function of NK cells in liver cancer lesion. In addition, curcumin can also decrease the levels of inflammatory factors (e.g., IL-6 and TNF-α) in mouse serum, and reduce immune injury caused by liver cancer (Jagetia and Aggarwal, 2007). It was found that curcumin exerted its antitumor effect by regulating various signaling pathways, including inhibiting tumor cell proliferation, invasion and migration and promoting apoptosis through down-regulating the expression of Bcl-2 family proteins, up-regulating the expression levels of p53 and Caspase-3, etc (Jagetia and Aggarwal, 2007; Zhai et al., 2020). Curcumin could inhibit the growth and proliferation of HT-29 colon adenocarcinoma cell line *in vitro*, through up-regulating the expression level of p53, Bax and Caspase-3, down-regulating the expression level of Bcl-2 (Yuandani et al., 2021). Curcumin may inhibit the growth of breast cancer cells by inhibiting the TLR4/TRIF/IRF3 signaling pathway and the secretion of inflammatory factors (Eskiler et al., 2019). Additionally, curcumin showed an important role in inhibiting lung squamous cancer cell metastasis and invasion by up-regulating the expression level of FOXA2, down-regulating the expression level of FOXA1, FOXA3 (Tang et al., 2018), matrix metalloproteinase (MMP) 2 and MT1-MMP (Liao et al., 2015) (Figure 4). Taken together, curcumin may play a therapeutic role in cancer by directly or indirectly regulating the function of the immune system. Although the results of these studies are relatively preliminary, they provide enlightenment for further exploring the immunomodulatory effect and mechanism of curcumin. Overall, curcumin is expected to be an effective drug for tumor immunotherapy (Paul and Sa, 2021).

### 3.2 Therapeutic effects and acting mechanisms of curcumol

Curcumol, a promising anticancer metabolite, disrupts several key pathways linked to inflammation and tumor growth, notably the NF-κB and MAPK pathways (Yang et al., 2021). It has demonstrated efficacy against solid tumors including those in the head, neck, colorectal region, breast, and lungs (Hashem et al., 2021). A specific analogue of Curcumol, T83, derived from Curcuma longae Rhizoma, enhances apoptosis and radiosensitivity by targeting the Akt/mTOR pathway and activating p38 MAPK, which diminishes the stability and function of Jab1 (Pan et al., 2013).

The metabolite’s mechanism involves curtailing processes crucial for tumor proliferation, metastasis, survival, invasion, and evasion of apoptosis (Hashem et al., 2021). For instance, in nasopharyngeal carcinoma and lung cancer, Curcumol has been observed to suppress tumor-promoting cytokines such as NF-κB, Bcl-2, and VEGF, while upregulating cytokines like PARP and AIF that promote apoptosis. It also arrests tumor cells in the G1 phase through the activation of p53 and pRB pathways, suppression of cyclinA1 and CtBP1, and upregulation of p21WAF1, p27KIP1, and CDK8 (Wei et al., 2019).

In a study by Yan et al. (2018), Curcumol administered at 15 μg/kg twice daily via gavage in a xenograft tumor mouse model led to significant tumor suppression. The treatment reduced TGF-β1 and N-cadherin levels while increasing E-cadherin, effectively blocking the epithelial-mesenchymal transition (Yan et al., 2018). Beyond its anticancer properties, Curcumol has shown potential in reducing metastasis and proliferation of prostate cancer cells by modulating lncRNA NR2F1-AS1/miR-145-5p22, inhibiting proliferation of HaCaT cells and the release of pro-inflammatory factors induced by interleukin-22. It also enhances the expression of SIRT1 mRNA in injured kidneys, thus diminishing NF-κB and NLRP3 levels and reducing IL-1β effects. Furthermore, it prevents apoptosis of hepatic stellate cells triggered by the PI3K/AKT/mTOR pathway, combating liver fibrosis and tumor angiogenesis. Additionally, Curcumol’s impact on the EGFR/PI3K/AKT and HIF-1α/VEGF pathways helps protect mouse models from cerebral ischemia-reperfusion injury by reducing oxidative stress and apoptosis in hippocampal neurons and inhibiting JNK1 activation (Chen et al., 2014; Wei et al., 2019; Hashem et al., 2021).

### 3.3 Therapeutic effects and acting mechanisms of β-elemene

β-elemene, a bioactive metabolite derived from Curcuma longae Rhizoma, has been recognized for its diverse biological activities, including anti-inflammatory, antioxidant, anti-cancer, and anti-microbial effects (Chen et al., 2021). Its mechanisms in cancer involve inducing cell cycle arrest, autophagy, apoptosis, and inhibiting cell migration and invasion (Bai et al., 2021). Additionally, β-elemene has been shown to enhance the effectiveness of chemotherapy and radiotherapy, suggesting its potential as an adjuvant therapy in cancer treatment (Pan et al., 2023).

The anti-cancer efficacy of β-elemene operates through various pathways, such as apoptosis induction, inhibition of cell proliferation and migration, and regulation of proteins related to cancer progression. Zhai B et al. (2019) highlighted the potential of β-elemene in combination with other chemotherapeutic agents to enhance their efficacy and reduce side effects (Zhai et al., 2019). For instance, Wang et al. (2018) demonstrated that β-elemene induced apoptosis in human cervical cancer SiHa cells in a dose-dependent manner at concentrations of 20, 30, and 40 μg/mL. The treatment altered the expression of key regulatory proteins, resulting in inhibited cell proliferation, G1 phase arrest, induced apoptosis, and reduced migration and invasion (Wang et al., 2018).

The molecular targets and signaling pathways influenced by β-elemene include PI3K/Akt/mTOR, NF-κB, MAPK/ERK, and Wnt/β-catenin (Qureshi et al., 2019). Moreover, β-elemene serves as a chemosensitizer in cancer treatments, potentially reducing drug resistance and enhancing overall therapeutic outcomes by affecting molecular targets such as hypoxia-inducible factor-1α (HIF-1α), DNA damage repair, and reactive oxygen species (ROS) generation (Tong et al., 2020).

Despite the promising results in preclinical and clinical studies, as noted by Jiang et al. (2017), critical analysis reveals several limitations in the research design of studies involving β-elemene (Jiang et al., 2017). Many studies lack robust control conditions and large, diverse sample sizes that could validate the generalizability of the findings. Additionally, the pharmacokinetic profiles of β-elemene are not well-documented, which raises concerns regarding its absorption, distribution, metabolism, and excretion in humans. Furthermore, most studies have focused on short-term outcomes without assessing long-term safety and efficacy, which are crucial for validating any potential clinical application. The variability in experimental settings and dosages also makes it difficult to standardize treatment protocols and to compare outcomes across different studies effectively.

To advance the therapeutic potential of β-elemene, future research must address these design flaws by implementing more rigorous, randomized controlled trials with standardized dosing regimens, comprehensive pharmacokinetic evaluations, and long-term safety and efficacy assessments. This will help establish a clearer understanding of the role of β-elemene in cancer therapy and its potential integration into standard cancer treatment protocols.

### 3.4 Therapeutic effects and acting mechanisms of curdione

Curdione, a prominent active metabolite found in Curcuma longae Rhizoma, exhibits a range of health benefits, including antioxidant, anti-inflammatory, anti-cancer effects, immune system regulation, and cardiovascular health improvement. According to basic research, curdione effectively reduces inflammation and inhibits the growth and spread of cancer cells, showcasing its therapeutic potential as a novel metabolite of turmeric (Wu and Tong, 2022).

In cancer research, curdione has been shown to affect the proliferation of the liver cancer HepG2 cell line through the VEGF/VEGFR2 signaling pathway. It also disrupts thrombin-induced platelet aggregation by regulating AMPK, which influences the vinculin/talin-mediated integrin signaling pathway. Moreover, curdione contributes to the reduction of hypertrophic scars by down-regulating the TGF-β1/Smads and PI3K/Akt/mTOR signaling pathways, effectively inhibiting human skin fibroblast cell proliferation and transformation, reducing collagen secretion, and promoting collagen enzymolysis (Rurou et al., 2021).

Additionally, curdione has shown cardioprotective advantages. For instance, it may help mitigate heart cell damage caused by doxorubicin—a common chemotherapeutic agent linked to heart failure—by activating the Nrf2/ARE signaling pathway, which is crucial for regulating cellular responses to oxidative stress (Liu et al., 2020). In studies of doxorubicin-induced cardiotoxicity in animal models, curdione demonstrated a promising ability to reduce inflammation, oxidative stress, and cell death in heart cells (Yarmohammadi et al., 2021). Specifically, Wu et al. (2019) found that in an experimental model using H9c2 cells, a dose of 100 μM curdione administered for 24 h, along with 1.25 μM doxorubicin for the same duration, activated Nrf2 and upregulated HO-1 expression, effectively combating cardiac toxicity. In summary, the potential therapeutic effects and mechanisms of action of curcumol, β-elemene, and curdione are outlined in Table 2, emphasizing their diverse impacts and the pathways they influence.

**TABLE 2:** Potential applications and possible mechanisms of action of curcumol, β-elemene, curdione.

Bioactive metabolites of Curcuma	Potential therapeutic effect		Pathways involved	Mechanism of action	References
Curcumol	Cancer	Solid tumors	Inhibiting the Akt/mTOR pathway	Promote apoptosis and radiosensitivity Reducing the stability and transcriptional activity of Jab1	Shehzad et al. (2012), Yang et al. (2021), Hashem et al. (2021)
			Activating the p38 MAPK pathway		
		Carcinoma and lung cancer	Activating p53 and pRB pathways	Inhibiting the expression of cyclinA1 gene Up regulating the expression of p21WAF 1, p27KIP1 and CDK8 genes	Yang et al. (2021)
		Prostate cancer	PI3K/AKT/mTOR signaling pathway	Inhibiting the level of NF-κB and NLRP3, reducing the effect of inflammatory factor IL-1β	Hashem et al. (2021), Chen et al. (2014)
β-elemene	Cancer		PI3K/Akt/mTOR, MAPK/ERK, NF-κB, and Wnt/β-catenin pathways	-Hypoxia-inducible factor-1α (HIF-1α), DNA damage repair and reactive oxygen species (ROS) generation -Inducing cell cycle arrest, apoptosis, autophagy, and inhibiting cell migration and invasion	Pan et al. (2023), Qureshi et al. (2019), Tong et al. (2020)
Curdione	Liver cancer		VEGF/VEGFR2 pathway PI3K/Akt/mTOR and TGF-β1/Smads signaling pathways	-Inhibit cell proliferation and transformation -Reducing collagen secretion and promoting collagen enzymolysis	Rurou et al. (2021)
	Cardioprotection		Nrf2/ARE signaling pathway	Reducing oxidative stress, inflammation, and cell death in heart cells	Liu et al. (2020), Yarmohammadi et al. (2021)

## 4 Enhancement of the bioavailability of active metabolites

The bioavailability of curcumin, despite its significant therapeutic potential, is challenged by several physiological barriers. Curcumin suffers from low bioavailability due to its poor absorption in the small intestine, rapid metabolism, and systemic elimination. It is also prone to auto-oxidation and degradation when bound to mucus in the gastrointestinal tract, which impedes uptake by epithelial cells (Stanić, 2017; Stohs et al., 2020). Additionally, factors such as the composition and metabolism of human intestinal flora are known to influence the bioavailability of curcumin (Anand et al., 2007; Scazzocchio et al., 2020). Heger et al. (2014) further elucidated that curcumin’s low oral bioavailability results from poor absorption, elimination via the gallbladder, and binding to enterocyte proteins, which may modify its structural integrity. Addressing these issues is critical in enhancing the bioavailability of curcumin.

To overcome these limitations, researchers have developed various strategies. These include modifying the molecular structure of curcumin, synthesizing new derivatives, and employing nanotechnology (Abd El-Hack et al., 2021; Tabanelli et al., 2021). For instance, nanotechnology has proven effective in preclinical and clinical studies for modifying curcumin’s bioavailability. Biocurcumax™, a curcumin-enhancing formulation, demonstrated significantly higher bioavailability compared to pure curcumin, with the relative bioavailability in the Biocurcumax group being nearly seven times that of a normal curcumin preparation (Antony et al., 2008; Tabanelli et al., 2021). Additionally, a nanoformulation of diethyl curcumin disuccinate (CDD) using chitosan/alginate nanoparticles (CANPs) has shown enhanced bioavailability and increased cytotoxicity to human hepatocellular carcinomas (HepG2) cells, indicating its potential for cancer treatment (Sorasitthiyanukarn et al., 2021).

Moreover, the use of whole plant extracts, which may offer synergistic effects from various metabolites, has shown promise. Rasoanaivo et al. (2011) found that whole plant extracts of Curcuma longae Rhizoma are more effective than isolated curcumin in reducing the growth and spread of the malaria parasite in both *in vitro* and *in vivo* models. These approaches not only enhance the clinical efficacy of curcumin but also guide further research on its bioavailability, paving the way for the development of curcumin-derived medicines and health products.

## 5 Limitations and future research need

The therapeutic promise of Curcuma longae Rhizoma’s active metabolites—curcumin, β-elemene, curcumol, and curdione—has been well-documented, showcasing significant anti-inflammatory, anticancer, antioxidant, and antimicrobial properties. However, despite the progress in identifying these benefits, a comprehensive understanding of the underlying mechanisms remains somewhat elusive. Current research provides a baseline understanding, but deeper insights are needed into how these metabolites interact with biological systems at molecular, cellular, and systemic levels. Future studies should aim to elucidate these mechanisms more precisely, improving our ability to use these metabolites effectively in targeted therapies.

In tandem with these scientific inquiries, there are also substantial opportunities for advancements in the methods used to extract and purify these bioactive metabolites. Traditional extraction techniques, while effective, often suffer from drawbacks such as low yield, high energy consumption, and potential degradation of sensitive metabolites. Emerging technologies, such as supercritical fluid extraction, ultrasonic-assisted extraction, and microwave-assisted extraction, represent the forefront of innovation in this area. These methods not only promise higher efficiencies and better yields but also align with the principles of green chemistry, reducing the environmental impact of production processes. Moreover, the integration of these advanced extraction techniques with a deeper mechanistic understanding could revolutionize the use of Curcuma longae Rhizoma in clinical settings. This holistic approach will not only optimize existing therapeutic applications but also potentially unveil new uses for these metabolites in medicine. As research continues to advance, it will be crucial to leverage both cutting-edge technology and rigorous scientific methods to fully harness the potential of Curcuma longae Rhizoma, setting a new standard for natural product research and its application in modern healthcare.

Despite the progress made, there are significant limitations that need to be addressed to fully leverage Curcuma longae Rhizoma’s therapeutic potential. Current studies primarily focus on isolated cellular models or animal studies, and there is a pressing need for robust clinical trials to validate these findings in human populations. Additionally, the variability in research methodologies—ranging from dosing regimens to the models used—hampers the ability to draw consistent conclusions across studies. Future research should focus on standardizing these elements to ensure reliability and comparability of results. Moreover, addressing the pharmacokinetics of these bioactive metabolites, especially their poor bioavailability and rapid metabolism, is crucial. Innovative delivery systems like nanotechnology could play a pivotal role in enhancing the clinical efficacy of these metabolites. Lastly, exploring the complex mechanisms through which these metabolites act on various diseases at the molecular level will be essential for developing targeted therapies. This approach not only promises to expand the therapeutic scope of Curcuma longae Rhizoma but also enhances our understanding of its role in disease management and prevention.

## 6 Conclusion

Curcuma longae Rhizoma is enriched with a multitude of active metabolites, and this review has focused on the four most extensively researched metabolites: curcumin, β-elemene, curcumol, and curdione. These metabolites have demonstrated a wide array of therapeutic effects, including antitumor properties, cardiovascular protection, anti-fibrotic actions, anti-inflammatory and analgesic effects, antiviral activities, glycemic control, and antioxidant capabilities. The pharmacological actions of these metabolites are manifested in several key areas: anti-inflammatory, antioxidant, anticancer, neuroprotective, and antimicrobial effects.

Curcuma longae Rhizoma’s metabolites target multiple biological pathways and exert their therapeutic effects through diverse mechanisms of action. Contemporary research has utilized advanced analytical methods along with metabolomics, transcriptomics, network pharmacology, and molecular docking techniques to elucidate the dose-response relationships of these active metabolites and their specific mechanisms of action against various diseases, including defining their safe dosage ranges. However, despite these advances, further research is necessary to deepen our understanding of the pharmacological effects and mechanisms of these active metabolites. Additionally, there is a critical need to enhance the bioavailability of these metabolites to maximize their therapeutic potential. Enhancing bioavailability will not only improve efficacy but also facilitate the broader development and clinical application of Curcuma longae Rhizoma, making it a more viable option in preventive and therapeutic healthcare settings.

## Abbreviations

TCM, Traditional Chinese Medicine; NF-κB, Nuclear factor kappa-B; MAPK, Mitogen-activated protein kinase; PPAR-γ, Peroxisome proliferator-activated receptor γ; IAV, Influenza A virus; PI3K, Phosphoinositide 3 kinase; Nrf2, Nuclear factor erythroid 2-related factor 2; HMG-CoA reductase, 3-hydroxy-3-methyl glutaryl coenzyme A reductase; SREBP1c, Sterol regulatory element-binding protein 1c; ChREBP, Carbohydrate response element-binding protein; CPT1, Carnitine palmitoyltransferase 1; ACAT, Acyl-CoA cholesterol acyltransferase; MCP-1, Monocyte chemotactic protein-1; IL-1β, Interleukin-1β; TNFα, Tumour necrosis factor-alpha; IL-6, Interleukin-6; COX2, Cyclooxygenase-2; MPO, Myeloperoxidase; TBARS, Thiobarbituric acid reactive substances; TGF-β, Transforming growth factor-beta; TBI, Traumatic brain injury; HAT, Histone acetyltransferase; HIF-1α, Hypoxia-inducible factor-1α; ROS, Reactive oxygen species.
